# Successful Use of Ertapenem for the Treatment of *Enterobacter cloacae* Complex Infection of the Central Nervous System (CNS)

**DOI:** 10.1155/2019/7021586

**Published:** 2019-11-05

**Authors:** Sunish Shah, Dayna McManus, Jeffrey E. Topal

**Affiliations:** ^1^Yale New Haven Hospital, Department of Pharmacy Services, New Haven, CT, USA; ^2^Yale School of Medicine, Department of Internal Medicine, Section of Infectious Diseases, New Haven, CT, USA

## Abstract

A 55-year-old female with a past medical history of cocaine use and hypertension was admitted for intracranial hemorrhage requiring right decompressive craniotomy with duraplasty. Due to persistent fevers, a head CT scan obtained on day 28 of hospitalization identified a low-density subgaleal fluid collection overlying the duraplasty. Aspiration of this collection was sent for culture which grew 2+ *Enterobacter cloacae* complex susceptible to sulfamethoxazole-trimethoprim (SMX-TMP), gentamicin, ciprofloxacin, and ertapenem. Based on these results, the patient was transitioned from empiric vancomycin and ceftazidime to SMX-TMP and metronidazole. Despite treatment with SMX-TMP and metronidazole, aspirated subgaleal collection cultures remained positive for *E. cloacae*. Intrathecal gentamicin was therefore added; however, repeat subgaleal culture collections remained persistently positive. Given the persistently positive subgaleal culture collections, the patient was transitioned from SMX-TMP and metronidazole to ertapenem. After transition to ertapenem, subgaleal cultures were sterilized and the patient's infection was resolved. This report suggests ertapenem may be a viable option for central nervous system infections; however, further study is needed.

## 1. Introduction

Infections in the subgaleal space are unusual and often result from trauma [1]. Subgaleal abscesses have typically been reported as polymicrobial with *Staphylococcus aureus* and *Streptococcus* spp., representing the predominant pathogens [1, 2]. Management consists of drainage of these abscesses in conjunction with systemic antimicrobial agents that have adequate CNS penetration [1]. A limited armamentarium of antimicrobials with sufficient CNS penetration exists, and these antimicrobials typically require frequent dosing [3].

Ertapenem is a broad-spectrum antimicrobial with a long serum half-life due to significant protein binding, which makes it the only carbapenem to allow for once daily dosing [4]. Compared with other carbapenems, ertapenem may be less likely to induce resistance in *Pseudomonas* given its lack of antipseudomonal activity [5]. To date, there are no published human reports of ertapenem use for the management of CNS infections. However, pharmacokinetic data from a rabbit model of bacterial meningitis suggest that ertapenem has adequate penetration into inflamed meninges [6]. We report a case of a subgaleal abscess caused by *Enterobacter cloacae* complex that was successfully managed with intravenous (IV) ertapenem.

## 2. Case Report

**Figure 1:**
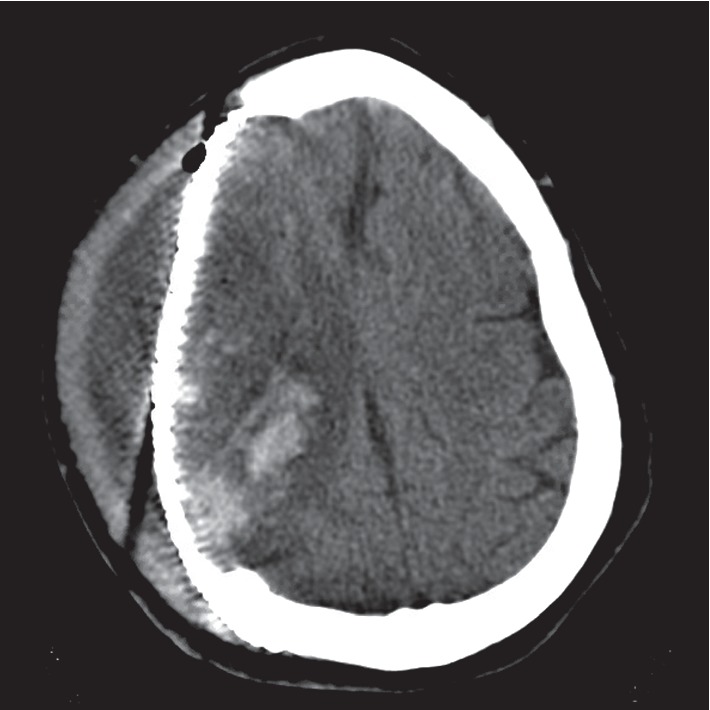
Head CT revealing an 18 mm subgaleal fluid collection overlying the duraplasty.

A 55-year-old female with a past medical history of cocaine use and hypertension was admitted for a large right basal ganglia hemorrhage with extension into all four ventricles requiring right decompressive craniotomy. Following a fever on day 23 of hospitalization, a head CT scan was obtained which revealed development of 3 new distinct parenchymal hemorrhages within the prior right cerebral hemorrhages. At this time, the patient's fever prompted initiation of empiric vancomycin and ceftazidime. On day 26 of hospitalization, the parenchymal hemorrhages were managed with neurosurgical debridement of the hemorrhagic brain with partial temporal lobectomy and duraplasty with titanium mesh placement since the patient failed to improve with medical management alone. A repeat head CT scan obtained on day 28 of hospitalization identified an 18 mm subgaleal fluid collection overlying the duraplasty noted in Figure 1. Given the persistent fevers, the subgaleal fluid collection was aspirated on day 30 of hospitalization which subsequently grew 2+ *Enterobacter cloacae* complex susceptible to TMP-SMX, gentamicin, ciprofloxacin, and ertapenem. Resistance patterns of the patient-specific *E. cloacae* isolated are described in Table 1. Cell count of the cerebrospinal fluid (CSF) identified 17,300 nucleated cells/*μ*L with 98% granulocytes, 35,000 red cells/*μ*L, 980 mg/dL of protein, and an undetectable glucose level.

**Figure 2:**
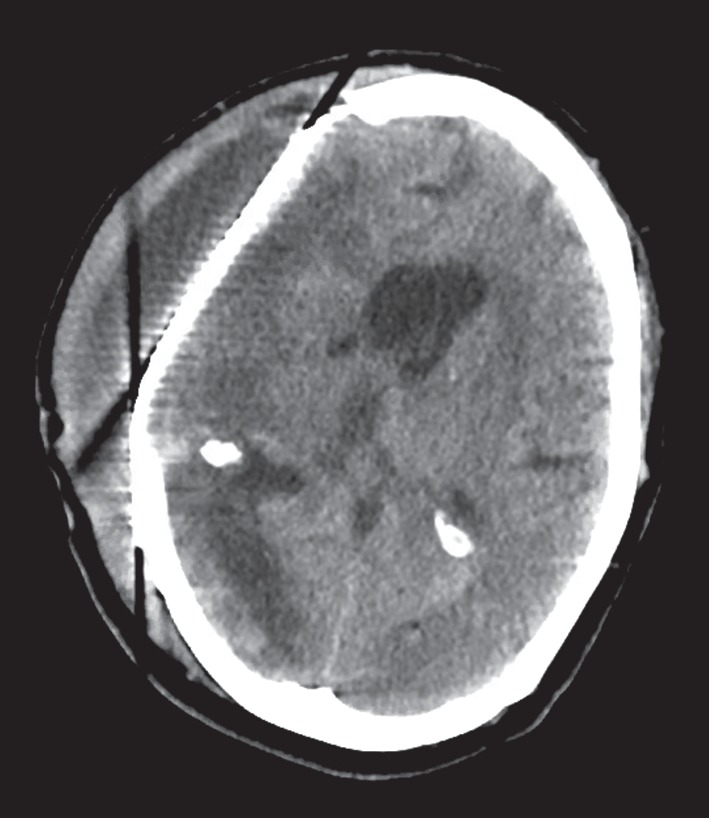
Repeat head CT was performed identifying an enlarging subgaleal fluid collection measuring 14 × 23 mm.

**Figure 3:**
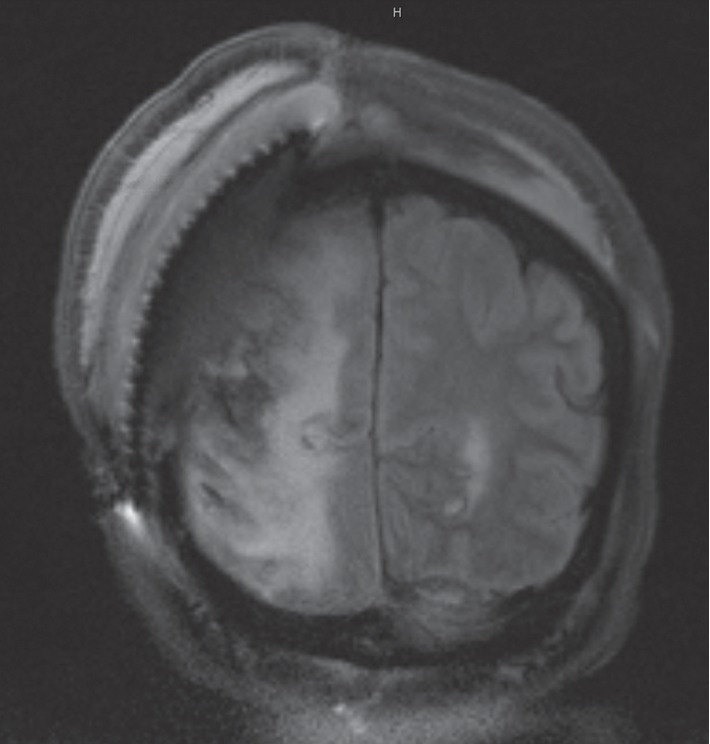
MRI of the brain demonstrating a decrease in the subgaleal fluid collection from 18 mm to 8 mm.

Given the susceptibilities, the patient was transitioned from vancomycin and ceftazidime to SMX-TMP (5 mg/kg of TMP) every 8 hours IV and metronidazole 500 mg IV every 12 hours. Since the patient continued to have persistent fevers, a repeat head CT scan was performed which identified an enlarging subgaleal fluid collection measuring 104 × 23 mm as noted in Figure 2. The neurosurgical team believed the risk of removal of the titanium mesh was much greater than the benefit given her poor surgical candidacy attributed to comorbidities. Furthermore, a future procedure would be needed following removal. Repeat subgaleal collection aspirations performed on day 35 of hospitalization remained persistently positive for *E. cloacae* prompting placement of a lumbar drain. Daily intrathecal gentamicin at a dose of 4 mg was started at day 36 of hospitalization and was administered daily through the lumbar drain. A follow-up head CT scan was performed and revealed no significant change in the subgaleal abscess size. Given the lack of improvement on imaging, repeat cultures of the subgaleal fluid collections were obtained and remained positive for *E. cloacae*. Repeat CSF cell count revealed 139,000 nucleated cells/*μ*L with 95% granulocytes, 351,000 red cells/*μ*L, 1,910 mg/dL of protein, and an undetectable glucose level. Given the persistently positive cultures and worsening CSF profile, after 11 days of SMX-TMP and metronidazole, the patient was transitioned to ertapenem 1 g IV daily. A CSF culture obtained 3 days after the initiation of ertapenem was found to be sterile. CSF cell count from this specimen revealed improvement and identified 112 nucleated cells/*μ*L with 83% granulocyte predominance, 7 red cells/*μ*L, 449 mg/dL of protein, and a glucose level of 32 mg/dL. An MRI of the brain obtained 7 days after ertapenem was initiated revealed a decrease in the subgaleal fluid collection from 18 mm to 8 mm as noted in Figure 3.

On day 50 of hospitalization, intrathecal gentamicin was stopped. The lumbar drain was removed after 2 weeks of therapy with ertapenem given achievement of a drainage rate of less than 10 mL/hour. After 16 days of ertapenem, the patient was transitioned to SMX-TMP for prolonged therapy given the presence of the titanium mesh. She was ultimately discharged to a rehabilitation facility after 77 days of hospitalization.

## 3. Discussion

To our knowledge, this is the first clinical case in which ertapenem was successfully utilized for the management of a CNS infection. Given *E. cloacae*'s high potential for inducible resistance to many beta-lactam antibiotics despite in vitro susceptibility, limited treatment options exist [7]. These treatment options were further limited given the need for an antimicrobial with adequate CNS penetration in our patient. Guidelines from the Infectious Diseases Society of America recommend TMP-SMX or meropenem for healthcare-associated ventriculitis and meningitis due to *E. cloacae* [3]. Although ciprofloxacin is suggested to have CNS penetration in some reports, treatment failure with ciprofloxacin has been observed for CNS infections due to the relatively low concentrations of drug in the CSF following systemic administration [8–10]. Both clinical data and experimental data have demonstrated adequate CNS penetration with TMP-SMX [11, 12]. Thus, it remains unclear why TMP-SMX failed microbiologically in this case despite in vitro susceptibility [11, 12]. However, it should be noted that consistent drainage was a key aspect of the patient's clinical success and may explain the microbiologic failure of TMP-SMX. The utility of intrathecal gentamicin in this case remains unclear; however, observational data have reported success of intraventricular gentamicin when used in combination with systemic antimicrobials [13]. On the other hand, bacterial abscesses are noted to have acidic environments which may render aminoglycosides ineffective [14, 15]. In our patient, the addition of intrathecal gentamicin did not result in neither microbiologic clearance nor radiographic improvement of the patient's infection.

Given microbiologic failure with TMP-SMX and intrathecal gentamicin in our patient, therapy with carbapenem was investigated. While meropenem has been extensively studied in patients with CNS infections, its use is limited by its frequent dosing and high propensity for inducing *P. aeruginosa* resistance given its broad spectrum of activity [16]. In addition to frequent dosing, higher doses of meropenem also need to be utilized to allow for adequate CSF penetration [16]. Use of ertapenem, a once daily antimicrobial, has been associated with a decreased incidence of carbapenem-resistant *P. aeruginosa* compared to meropenem or imipenem [5]. Compared with other carbapenems, ertapenem also has increased lipophilicity and a larger volume of distribution due to a metasubstituted benzoic acid group at position-2 [17, 18]. These important structural and kinetic differences would suggest improved CNS penetration. Given the likely improved CNS penetration over other carbapenems, reduced potential to select for resistant organisms, and streamlined once daily dosing, ertapenem was deemed the most appropriate carbapenem to initiate for our patient. Although there is a lack of clinical data for the use of ertapenem for the treatment of CNS infections, a prior study assessed the pharmacokinetics of ertapenem in rabbits inoculated with *S. pneumoniae* in the cisterna magna [6]. At a dose of 60 mg/kg, CSF ertapenem levels remained above the MIC throughout the entire treatment period. The CSF penetration to serum ratio of ertapenem reached 7.1% and 2.4% into inflamed and noninflamed meninges, respectively. More importantly, ertapenem sterilized the CSF of all 10 rabbits inoculated with penicillin-susceptible *S. pneumoniae*.

A limitation of this report is that we were unable to obtain levels of ertapenem in our patient given that assays to detect CSF levels are not commercially available. However, the clinical efficacy of ertapenem was demonstrated with sterilization of the CSF cultures, improvement of CSF cell counts, and radiologic improvement. Furthermore, the patient had clinical neurologic improvement following therapy with ertapenem. While our patient did not experience adverse effects attributed to ertapenem, seizures and encephalopathy have been rarely reported with ertapenem therapy [19, 20]. However, unlike encephalopathy, seizures have been reported as a class effect amongst the carbapenems with the FDA reporting that imipenem, meropenem, and ertapenem are associated with a seizure risk of 0.4, 0.7 and 0.5%, respectively [20].

In conclusion, we describe a case of a subgaleal abscess with ventriculitis caused by *E. cloacae* complex that was successfully managed with ertapenem and placement of a lumbar drain. Given the limited number of antimicrobials that can be utilized for CNS infections, investigating the use of other antimicrobials with CNS penetration is needed. Our case report suggests further evaluation of ertapenem for the management of CNS infections is warranted.

## Figures and Tables

**Table 1:** Resistance pattern of the *E. cloacae* isolated.

	Ciprofloxacin	Gentamicin	SMX-TMP	Ertapenem
*Enterobacter cloacae*	S	S	S	S

SMX-TMP: Sulfamethoxazole-trimethoprim.
